# Validation of a novel medical device (Chloe SED®) for the administration of analgesia during manual vacuum aspiration: a randomized controlled non-inferiority pilot study

**DOI:** 10.3389/fpain.2024.1326772

**Published:** 2024-09-18

**Authors:** Aparna Ramanathan, Karlheinz Tondo Samenjo, Robert C. Bailey, Javan Imbamba, Stella Odenyo, Erin Koksal, Jan Carel Diehl, Jackton Omoto, Stephen Gwer

**Affiliations:** ^1^ National Center for Advanced Pelvic Surgery, Department of Obstetrics and Gynecology, Medstar Washington Hospital Center, Georgetown University, Washington, DC, United States; ^2^ Nyanza Reproductive Health Society, Kisumu, Kenya; ^3^ Faculty of Industrial Design Engineering, Delft University of Technology, Delft, Netherlands; ^4^ Department of Epidemiology, University of Illinois at Chicago School of Public Health, Chicago, IL, United States; ^5^ Department of Obstetrics and Gynaecology, Maseno University, Kisumu, Kenya; ^6^ Independent Researcher, Cambridge, MA, United States

**Keywords:** paracervical block, safe abortion, anesthesia, human rights, miscarriage, novel technology, clinical trial, syringe extension device

## Abstract

**Introduction:**

Millions of women worldwide annually undergo manual vacuum aspiration (MVA) with no pain medication, which is a violation of their basic human dignity. We designed a novel device (Chloe SED®) to administer paracervical block (PCB) during MVA in countries where pain medication is not typically given due to the high cost of the necessary tools.

**Methods:**

We conducted a single-blinded, randomized controlled non-inferiority trial including 61 patients at two hospitals in Kisumu, Kenya, to validate Chloe SED® for administration of PCB during MVA. PCB administered with Chloe SED® was compared to PCB administered with a standard spinal needle. Patients requiring MVA were block randomized in blocks of six, each provider completing six PCBs—three with the Chloe SED® and three with the standard spinal needle. The trial was registered with the Kenya Pharmacy and Poisons Board, ECCT/19/03/01 (https://ctr.pharmacyboardkenya.org/applications/index/protocol_no:RUNDVC8xOS8wMy8wMQ__/filter:/investigator:/sites:/pages:5/start_date:/end_date:/disease_condition:/users:/ercs:/stages). An intention-to-treat analysis was completed. The primary outcome was the non-inferiority of the pain score during uterine evacuation with a non-inferiority margin of 2 points on an 11-point numerical rating scale. Secondary outcomes included the non-inferiority of the pain score at four other time points and patient satisfaction.

**Results:**

Chloe SED® showed non-inferiority of the primary outcome with a mean pain score during evacuation of 3.8 [90% confidence interval (CI): 3.1–4.6] compared with the spinal needle at 4.1 (90% CI: 3.5–4.7). Non-inferiority of the pain score was shown at all time points. Most patients expressed a desire for the continued use of the device to administer PCB for MVA. No adverse events were noted.

**Conclusion:**

In summary, the Chloe SED® appears non-inferior to the spinal needle and desirable for the administration of PCB during MVA.

## 1 Introduction

Approximately 75 million women globally experience pregnancy loss each year (1). Manual vacuum aspiration (MVA) is a common method for the treatment of first-trimester pregnancy loss worldwide. It is arguably the least expensive and most expedient method of evacuating the uterus, associated with fewer complications and side effects than dilation and curettage (2). MVAs are widely used in low-resource countries, are often performed by nurses or midwives, and do not require electricity or an operating theater. Currently, more than 300,000 women undergo MVAs in Kenya annually (3, 4).

MVAs cause considerable pain from the manipulation of the cervix and uterine suction (2). They are often performed in Kenya (and elsewhere in low-resource settings) without any analgesia (5, 6). The reasons cited for these pain control gaps in Kenya include the belief of the surgical provider that pain medication is unnecessary; the lack of availability of medication and equipment; and inadequate training in the provision of pain control including paracervical block (PCB) (6). Importantly, in a study of Kenyan women, all women who underwent MVA without pain medication desired it for future procedures, even at additional cost (6). Similarly, in an Ethiopian study, fear of pain was a factor for women in choosing medical over surgical treatment for miscarriage (7).

In March 2022, the WHO published new safe abortion guidelines recommending that PCB be used universally for pain control during MVA (8). This marks a significant change from the previous guidance, which did not specifically recommend any analgesics (9). However, clinics in low-resource settings face barriers in following these recommendations due to cost and supply chain interruptions in sourcing the spinal needles or needle extenders required for PCB.

**Figure 1:**
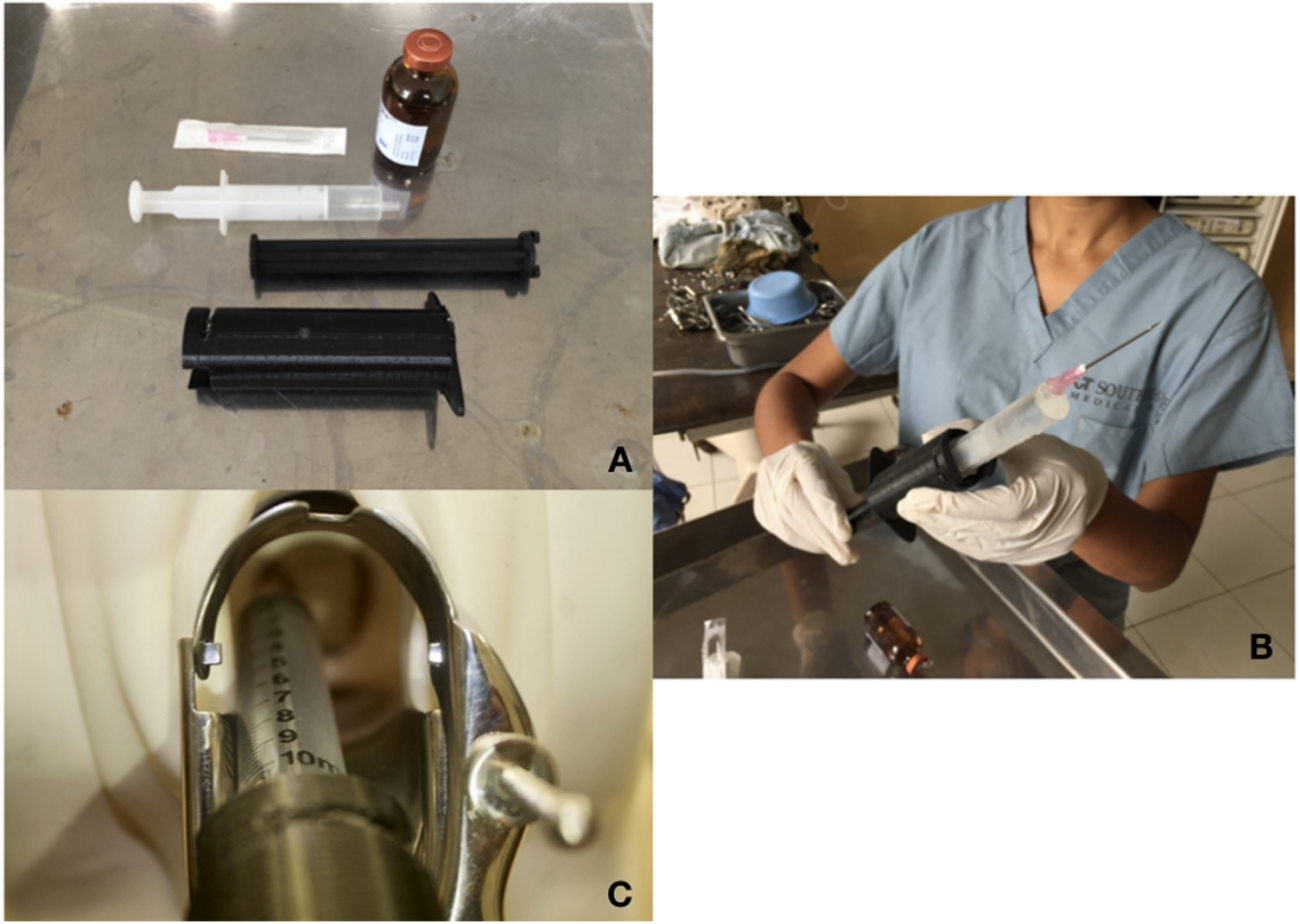
Chloe SED®. Chloe SED® is comprised of two components that attach to the syringe body and the syringe plunger of a 10 cc syringe. Components and syringe are shown disassembled **(A)** and assembled **(B)**, Chloe SED® is also demonstrated on a pelvic mannequin **(C**).

We have developed a novel, reusable, low-cost syringe extension device (SED), named Chloe SED®, that attaches to a 10-cc syringe to provide the additional length required to administer a PCB with a standard-length 21-gauge needle (Figure 1). The device is designed to be reused multiple times after sterilization, taking into account environmental sustainability issues and moving away from the use-dispose approach currently practiced in the healthcare sector. Our team has previously published a paper outlining our context-driven approach to the design of this device and newer versions (10). Chloe SED® has the potential to expand access to humane pain relief for women requiring MVA and even other gynecologic procedures such as excision treatment of cervical pre-cancer, diagnostic uterine curettage, and intrauterine device insertion. The primary objective of this study was to validate the functionality of Chloe SED® for the provision of PCB during MVA in a pilot study. Functionality was assessed via measurement of patient pain scores during MVA utilizing either Chloe SED® (experimental arm) or a standard spinal needle (control arm) to administer PCB.

## 2 Materials and methods

### 2.1 Study design

We conducted a single-blinded, randomized controlled non-inferiority trial at Jaramogi Oginga Odinga Teaching and Referral Hospital (JOOTRH) and Kisumu County Hospital (KCH) in Kisumu, Kenya, from September 2019 to January 2021. It was a mixed methods study that included both quantitative and qualitative data collection in a convergent parallel design.

### 2.2 Participants

Licensed Kenyan Medical Officers (MOs) or Clinical Officers (COs) performing MVAs at either of the two sites were invited to enroll in the study. As the assignment of MOs and COs to the gynecology ward occurs on a rotational basis, providers were approached once they started their rotation on the gynecology ward by a member of the study team who described the study’s aims and procedures. Following all questions and a discussion regarding the study, interested MVA providers consented to enroll. Our major eligibility criteria for providers were English-speaking providers over the age of 18 who were experienced with the provision of MVAs.

Participants were recruited from patients coming to the health facility who required uterine evacuation for spontaneous or induced abortion. Once a patient was determined to be clinically eligible for MVA by a recruited provider and elected to have this treatment, that patient was invited to participate in the study. The eligibility criteria for patients were aged 18 years and older; evaluated by a recruited provider to be eligible for MVA; and fluent in English, Swahili, or Luo. Exclusion criteria were any contraindication to lidocaine including known hypersensitivity, infection in tissue adjacent to the proposed site of injection (including uterine and cervical infection), concomitant anticoagulation therapy or reported abnormal bleeding tendency, severe anemia, or heart disease. Severe anemia was defined as per the WHO as anemia associated with symptoms of fatigue, weakness, dizziness, and drowsiness, or a known hemoglobin concentration of less than 7.0. All provider and patient participants gave written informed consent.

### 2.3 Randomization and masking

After patients completed their written informed consent, they were randomized to receive PCB with either the Chloe SED® experimental device or with a standard spinal needle (control arm). One of the off-site study investigators, SG, created a computer-generated 1:1 randomization scheme in blocks of six. A separate investigator, AR, concealed the randomization in a series of numbered envelopes for each block. A research assistant (RA), JI, enrolled participants, assigned them to the trial groups using the sequential envelope numbers, and completed the data collection. Given that the Chloe SED® and spinal needle are different in appearance, the providers and research assistant could not be blinded to the treatment arm assignment. As patients were positioned in the lithotomy position for this gynecologic procedure, they were blinded to treatment arm assignment and the instruments were kept out of view. Study team members analyzing the data were not blinded to the group assignments.

### 2.4 Description of the Chloe SED® intervention

Patients randomized to the experimental arm received PCB with the Chloe SED® experimental device. Chloe SED® was designed by medical providers and design engineers familiar with the local context. It has one or two components (depending on the design version) that attach to a 10 cc syringe body and plunger. The model tested in this study has two components: one that attaches to the body and a second that attaches to the plunger. They act to extend syringe length such that administration of a PCB is possible using a standard-length 21-gauge needle. These 10 cc syringes and 21-gauge needles are widely available in Kenya at all health facility levels. The syringe extension device is made of plastic and can be sterilized with locally available glutaraldehyde in a similar manner to the Karman cannulas that are currently used for MVA, requiring no additional equipment at the health facilities where MVAs are completed. The Chloe SED® experimental devices used in this study were manufactured at AB3D 3D Printing in Nairobi, Kenya. They were made from polylactic acid (PLA) plastic.

### 2.5 Description of the control arm intervention

Patients randomized to the control arm received PCB with a 22-gauge spinal needle. The needles are single-use and were disposed of after use. The spinal needles used in the study were purchased within Kenya at local medical supply shops. Neither 21-gauge spinal needles nor 22-gauge standard-length needles were available in Kenyan medical supply shops. Therefore, the needles selected were the closest to the same gauge available and represent the needles commonly used in medical practice.

### 2.6 Procedures

Each provider who enrolled in the study participated in a semi-structured interview prior to the onset of any procedures about their experience with MVA and their perceptions about pain control. Each provider was then trained in the use of the Chloe SED® experimental device by one of the two principal investigators, AR and SG, who invented the device and could instruct on device use. Each provider also completed a refresher training on PCB administration. A second interview was conducted with each provider after the completion of their six procedures about their experience using Chloe SED® compared with the spinal needle.

**Figure 2:**
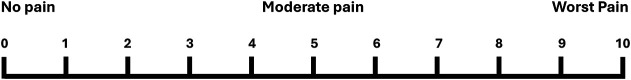
Numerical rating scale. Instructions for patients as administered by the study RA: Using this scale from 0 to 10, where 0 is “no pain,” 5 is “moderate pain,” and 10 is the “worst pain imaginable,” how much pain are you feeling right now?

When a provider evaluated a patient who was clinically eligible for MVA and elected to have this procedure, they contacted the study RA who administered the informed consent process. Patient participants then completed an initial face-to-face semi-structured interview about their experience with and perceptions about MVA. Following this, the provider completed the patient's MVA procedure with PCB. The PCB was administered per the training guidelines published by Ipas, an international organization that works globally to advance reproductive justice. Following Ipas guidelines, each patient received 200 mg of plain lidocaine during PCB, administered as either 20 cc of 1% lidocaine or 10 cc of 2% lidocaine depending on which formulation was available in the clinic at the time of the MVA. A small amount of lidocaine was injected at 12 o’clock to facilitate tenaculum placement with the remainder of the lidocaine equally distributed at 2, 4, 8, and 10 o’clock at the cervicovaginal junction (11). Patient pain level was assessed by the RA using an 11-point numerical rating scale (NRS) at five time points: just before the onset of the procedure, at the time of injection of the paracervical block, during cervical dilation (if dilation was required to complete the MVA), during the uterine evacuation, and 30 min post-procedure (Figure 2). Following the MVA, the patient completed a second semi-structured interview about their experience of the procedure. All interviews were conducted by a study RA. The interview guide was structured as multiple choice and Likert scale questions with opportunities to probe ideas presented at greater depth.

### 2.7 Outcomes

Our primary outcome was the comparison of pain scores on the 11-point NRS during uterine evacuation between patients receiving PCB with the Chloe SED® vs. with the spinal needle. Participants defined their pain score as an integer between 0 and 10, inclusive. Secondary outcomes included pain scores at the four other time points. Other secondary outcomes included: any adverse event, data on patient and provider experiences and perceptions, use of additional pain medications, patient satisfaction with pain management, and provider feedback on Chloe SED® design and usability compared with the spinal needle.

A core outcome set (COS) was not used in the design of this trial as a COS did not exist at the time of study design. When comparing our measured outcomes to those reported in a recent 2021 COS for general abortion research, outcomes that pertained to MVAs were assessed (12). No patients or members of the general public were involved in the design of this trial.

### 2.8 Statistical analysis

Non-inferiority testing was used for quantitative analysis of the primary outcome. We powered the study to detect a difference of 2 points on the NRS, as a change ranging from 1.3 to 2 points on this scale has been previously considered to be a clinically meaningful difference in pain level (13, 14). As we were only interested in non-inferiority and not equivalence, the sample size calculation was based on a one-tailed alpha of 0.05. A sample size of 28 patients per group provided 80% power to detect a 2 point difference based on a mean pain level of 6 on an 11-point NRS with a standard deviation of 3. Mean pain scores cited in previous studies ranged from 5.4 to 6.3 with the standard deviation ranging from 2.3 to 3.2 (15–17). To facilitate an equal number of patients being recruited by each of the 10 providers, we planned to recruit 60 patients, 30 in each arm. The mean and 90% confidence intervals (CIs) of the pain scores were calculated and the significance of the difference between the arms was estimated by a *t*-test. Pain level comparisons at the other four time points were compared in a similar fashion. Descriptive statistics were used for analysis of other secondary outcomes including patient satisfaction with the procedure, ease of use of the device, and incidence of adverse events.

Microsoft Access 2000 was used for data entry. Data were then exported into Stata 17.0 for statistical analyses. We analyzed our primary cohort using an intention-to-treat approach.

A Data Safety Monitoring Board (DSMB) comprised of three individuals with no conflict of interest monitored the study data. At the study midpoint (when five providers had completed the study with 30 patients), a qualitative assessment of the data was undertaken specifically looking for adverse events. There being none, the trial was continued.

### 2.9 Role of the funding source

This study was funded by the Department of Obstetrics and Gynecology at the University of Illinois at Chicago. The funder of the study had no role in study design, data collection, data analysis, data interpretation, or writing of the report.

## 3 Results

**Figure 3:**
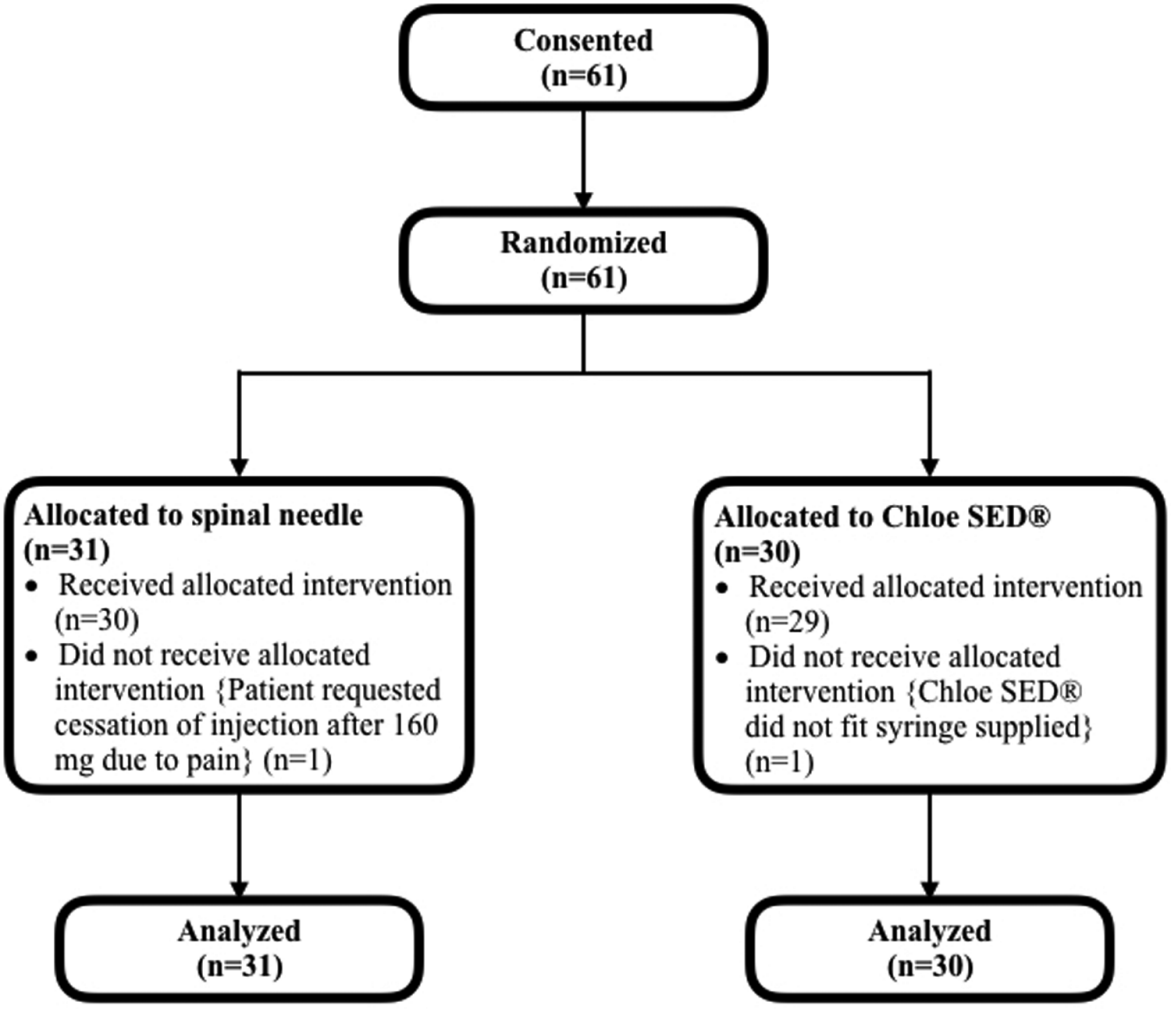
CONSORT (Consolidated Standards of Reporting Trials) flow diagram.

Between September 2019 and January 2021, 61 patients were recruited and randomized (31 to spinal needle and 30 to syringe extender) across the two facilities. One provider left the study after the enrollment of a single patient due to new employment in another city, so that block had only a single participant. This patient was not excluded and was retained in the syringe extender arm as per their randomization. In one block of six patients, there were four randomized to the spinal needle arm and two to the syringe extender arm due to an error in the creation of one randomization envelope. In one case, the syringe extender was noted not to fit the available syringe and was unable to be used for the paracervical block. In this case, a spinal needle was used to administer the block instead. This participant kept their assignment in the syringe extender group for the purpose of intention-to-treat analysis. One patient in the spinal needle arm requested cessation of PCB after 160 mg of lidocaine had been injected due to the pain from the injection. This patient kept their assignment in the spinal needle group for the purpose of intention-to-treat analysis. All other randomized patients received treatment per protocol (Figure 3). In total, 11 providers were recruited to participate in the study. Baseline patient participant characteristics did not differ between groups (Table 1). The median age of the participants was 26 (IQR 22–32). Most (67.2%) had received secondary schooling, had never before had an MVA (90.2%), and were multiparous (67.2%). The mean age of gestation for completion of the MVA was 10.0 weeks, with a minority (16.7%, 12.9%) of procedures in each group being completed for retained products of conception.

**Table 1:** Participant characteristics.

Characteristics	Spinal needle (*n* = 31)	Chloe SED® (*n* = 30)	Total (*n* = 61)
Age (years)	25 (22.5–29.5)	28 (22–32)	26 (22–32)
Parity			
Nulliparous	9 (29.0)	8 (26.7)	17 (27.8)
Parous	22 (71.0)	19 (63.3)	41 (67.2)
Obstetric history			
Prior vaginal deliveries	21 (67.7)	17 (56.7)	38 (62.3)
Prior MVA	3 (10.0)	3 (10.0)	6 (9.8)
Education			
None	1 (3.2)	0 (0.0)	1 (1.6)
Primary school	10 (32.3)	9 (30.0)	19 (31.1)
Secondary school	15 (48.4)	15 (50.0)	30 (49.2)
University or beyond	5 (16.1)	6 (20.0)	11 (18.0)
Gestational age (weeks)	9.4 (SD 3.0)	10.8 (SD 3.1)	10.0 (SD 3.1)
Procedures for retained products of conception	4 (12.9)	5 (16.7)	9 (14.8)

Data are median (interquartile range 1, 3), *n* (%), or mean (SD). Median was used for age; *n* (%) was used for parity, obstetric history, education, and procedures for retained products of conception; mean (SD) was used for gestational age.

**Figure 4:**
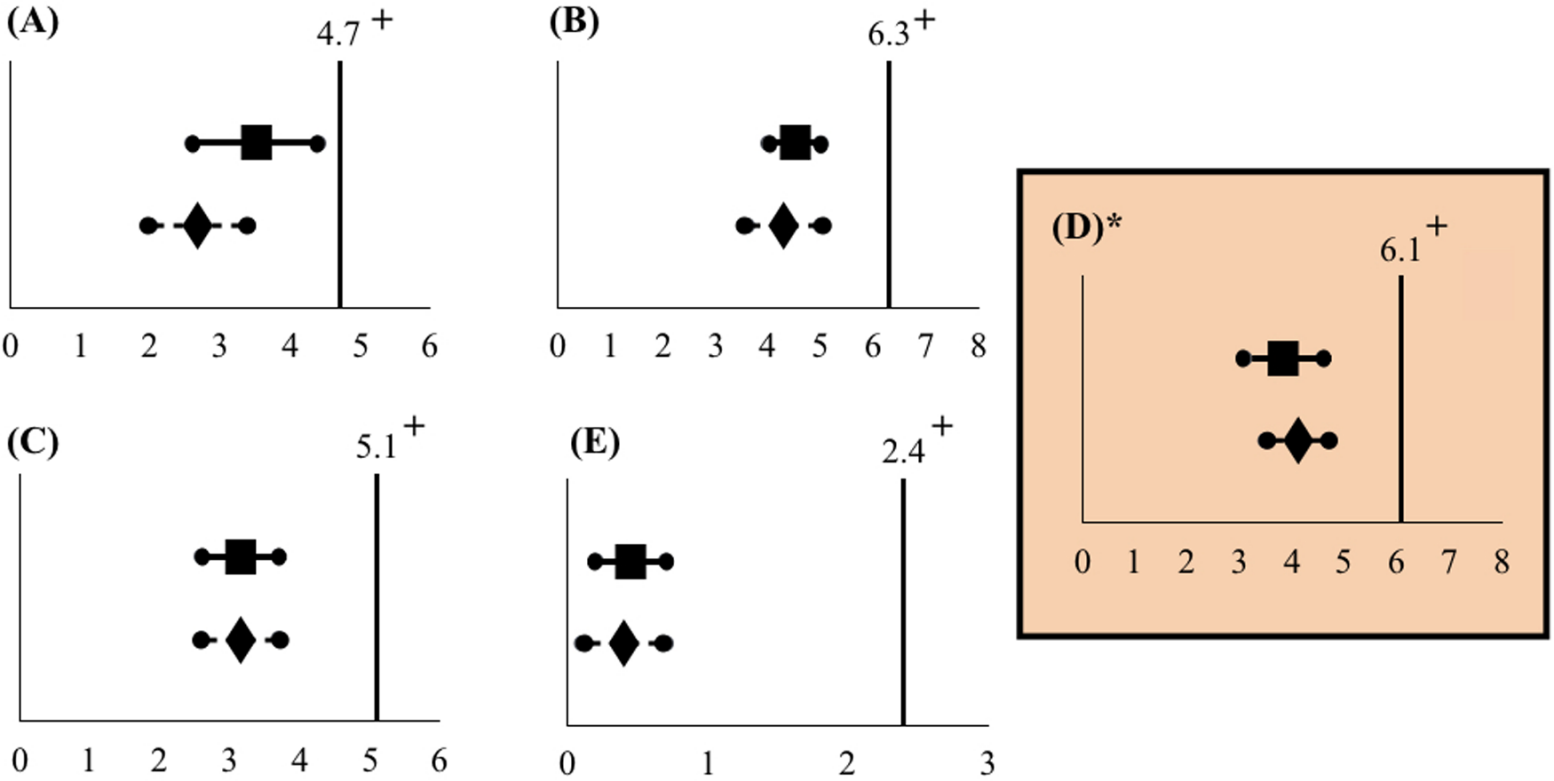
Mean pain scores in relation to the non-inferiority limit (intention-to-treat analysis). Mean pain scores and 90% confidence intervals do not cross the non-inferiority limit at any of the five time points: **(A)** Before MVA, **(B)** during injection of PCB, **(C)** during cervical dilation, **(D)** during uterine evacuation, and **(E)** 30 min after the procedure. The non-inferiority limit was set at 2 points on the NRS. *Primary outcome. 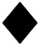 Spinal needle mean pain score. +Non-inferiority limit. 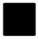 Chloe SED® mean pain score.

The intention-to-treat outcomes for NRS during uterine evacuation (primary outcome) and at four other time points including before the MVA, during injection of PCB, during cervical dilation, and 30 min following the procedure (secondary outcomes) are summarized in Table 2 (Figure 4). Non-inferiority of Chloe SED® for administration of PCB was found at all time points; the upper bound of the 90% CI was less than the 2 point difference set as the non-inferiority margin. No adverse events were reported. In one case, a finger pad on the Chloe SED® broke after the administration of the PCB and that case was completed. This breakage did not result in any injury to the patient or provider.

**Table 2:** Primary and secondary outcomes (intention-to-treat analysis).

Pain score timepoints	Spinal needle (*n* = 31)	Chloe SED® (*n* = 30)
Before MVA	2.7 (2.0–3.4)	3.5 (2.6–4.4)
During injection of PCB	4.3 (3.6–5.0)	4.5 (4.0–5.0)
During cervical dilation	3.1 (2.6–3.7) *n* = 28	3.1 (2.6–3.7) *n* = 26
During uterine evacuation^a^	4.1 (3.5–4.7)	3.8 (3.1–4.6)
30 min after procedure	0.4 (0.1–0.7) *n* = 30	0.4 (0.2–0.7)

Data are mean pain scores (90% confidence interval) on an 11-point NRS. Not all MVAs required cervical dilation to be completed. One patient did not report a pain score 30 min post-procedure.

^a^ Primary outcome.

We also collected pre-procedure and post-procedure semi-structured interview data from both patients and providers. These data were analyzed using descriptive statistics. All enrolled patients and providers completed both interviews. A small number of patients (three in each arm) had previously experienced an MVA outside of this study. In the pre-procedure interview, five of the six (83.3%) patients reported not receiving any pain medication during their previous procedure and these five patients noted that they were unhappy with their previous experience. One patient said, “It [MVA] hurts, it is disgusting. But it is better than oral medication.” None of the six patients were given any choice about whether or not to receive pain medication. In the total cohort of 61 patients, all were asked what was the most concerning aspect of the MVA procedure for them; 30 (49.2%) reported being most concerned about procedure pain. This was the most common response given ranking over other concerns including medical risks of the procedure (3.3%), anxiety or fear of the unknown (19.7%), and fear of passing out (1.6%).

In contrast, during the post-procedure interviews, 60 out of the 61 patients (98.4%) noted that the procedure was satisfactory or tolerable. One person (1.6%), who was in the spinal needle group, reported unhappiness with the procedure due to inadequate pain control. In total, 58 of the 61 patients (95.1%) would want to receive PCB for a future MVA procedure.

In the provider post-procedure semi-structured interviews, 7 out of 10 providers reported that it was easy or very easy to use Chloe SED® for PCB. Three providers described the ease of use as moderate. In the spinal needle arm, 6 out of 10 providers reported that it was easy or very easy to use the spinal needle for PCB with 4 providers describing the ease of use as moderate. One provider said, “It [Chloe SED®] is efficient in administering the block; easy to assemble and reuse after cleaning.” All providers (100%) noted that they would use Chloe SED® if it became available in the future. A detailed qualitative analysis of patient and provider interviews is discussed in a separate paper which is currently under review.

## 4 Discussion

### 4.1 Main findings

This study found that Chloe SED® was non-inferior to the standard spinal needle in the administration of PCB at both the primary time point (during uterine evacuation) and at all secondary time points. The mean pain scores in both arms of this study were lower than those previously reported in the literature, with a similar standard deviation of 2.3 points on the NRS (18).

### 4.2 Interpretation

Manual vacuum aspiration in Kenya and in other low- and middle-income countries is commonly performed without the administration of any pain medication whatsoever. Patients receive what is called “local vocal” or “keep quiet” anesthesia, which is the presence of a support person to issue words of comfort. While this presence of support is certainly important, it cannot be considered adequate or humane pain management for all women and in fact does not measure up to the standard of care seen in high-income countries, where non-steroidal anti-inflammatory drugs, PCB, anxiolytics, and moderate sedation are commonly provided for MVAs (19, 20).

In busy Kenyan clinics and hospitals, where women wait in line for MVAs, we have seen women who are actively bleeding and in need of medical care leave treatment wards upon hearing the screams coming from the treatment room as the woman ahead of them receives an MVA with no pain medication. The desire for more choice and autonomy in pain management is borne out in our study results as well, with a majority of women citing pain as their primary concern in having an MVA and greater than 95% of women desiring PCB for any future MVA procedure. Lack of humane pain control is an unacceptable limitation of a patient's right to access safe, quality medical care.

Several barriers to the use of PCB have been cited, including inaccurate beliefs among providers that pain control is not necessary for an MVA, lack of adequate medications and tools, and lack of training in providing PCB (6). With the successful results noted in this pilot study, we aim to alleviate one of these barriers—lack of access to tools required for administration of PCB. Chloe SED® is a novel and reusable device. With a projected cost at scale of 5 USD and a projected lifespan of 400 procedures, the Chloe SED® would reduce the cost of administration of PCB by greater than 90% compared to the use of single-use spinal needles. The cost of a standard 21-gauge needle is 0.01 or 0.02 USD per needle. This makes the incremental cost per procedure of Chloe SED® approximately 0.03 USD compared to 1–2 USD for each spinal needle procured, which is a cost savings of 97%–99% per procedure. Furthermore, 1–2 USD would easily translate to a full day's wages when that cost is transferred to the patient. The Chloe SED® would be a more affordable alternative.

As is largely the case for family planning services worldwide, the battle fought over the past 50 years has been about access to safe, affordable care and services. In Kenya, as well as in several other countries, there have been major victories in care access with the introduction of MVA and the ability to move procedural abortion care from hospitals to outpatient clinics. However, low-quality or inhumane treatment does not constitute meaningful access to care. The Lancet Commission recently stated that metrics reporting service quantity are meaningless if those services are not of high quality and do not fulfill a basic right to humane care (21). Chloe SED® has the potential to empower marginalized women to access respectful family planning services and to enable clinics to comply with the WHO's most recent safe abortion guidance, recommending PCB with every MVA performed (8).

### 4.3 Study strengths and limitations

Our clinical trial has several strengths related to study design. It was a randomized, multi-center trial. Patients were blinded to the study intervention, which should have reduced bias in the reporting of pain scores. Due to the short duration of PCB and lack of need for long-term follow-up after administration of the block, all patient data were complete with no loss to follow-up. Previous studies of PCB efficacy have been heterogeneous in methodology. Our study had a comprehensive methodology with a standardized method for PCB application and measurement of pain scores at five different time points, which is an improvement on the previously existing data. The Chloe SED® device is novel and there are no other studies testing this device or anything of a similar design. Our study also acknowledged that patient autonomy and preference are critical in the development of an ideal pain control strategy for MVA. As such, patient experiential and satisfaction data was collected.

Our trial has several limitations. As a pilot study, the sample size is small and cannot provide a comprehensive review of safety and adverse events. In addition, data regarding the number of patients who were approached by the study team and refused participation or who were unable to consent due to not meeting inclusion and exclusion criteria was not collected. Although we estimate based on case volume at the two sites that the rate of refusal or exclusion was less than 15%, we did not collect this information during the trial and are relying on data collected by the hospital wards for their own reporting purposes. Third, due to the difference in appearance between the spinal needle and Chloe SED®, the study providers were unable to be blinded. This could introduce bias in the administration of the PCB or evaluation of pain. The statistical analysis of pain scores was also completed unblinded, which may have introduced bias. Since the procedure was standardized and monitored by research assistants who are proficient in the provision of PCB, this type of bias is unlikely to have had a major effect on the results. Fourth, for the purposes of this pilot study, the higher end of the range of values noting a clinically significant difference in pain score was chosen for the non-inferiority limit. Based on the findings of this study, which show that the Chloe SED® is functional for administering PCB and that conduct of an RCT for this novel device is feasible in our target population, we are conducting a larger clinical trial for further assessment with a more narrowly defined non-inferiority limit. Fifth, we know that reporting of patient satisfaction in abortion studies tends to be high, likely because access to procedures is difficult and patients are generally thankful to receive medical treatment. However, albeit with a very small sample size, we do see a trend in greater satisfaction than among patients who previously experienced MVA without PCB. Finally, we know that a numerical unidimensional scale such as the NRS does not adequately capture the complex biopsychosocial experience of pain. However, it is a validated quantitative tool in pain assessment that we felt would be adequate in measuring differences between the two tools in their efficacy of administering PCB.

## 5 Conclusion

In summary, the Chloe SED® is non-inferior to the standard spinal needle in the administration of PCB for a difference of 2 points on the NRS. Further study is needed in larger sample sizes to further demonstrate safety and efficacy at a narrower non-inferiority margin. The Chloe SED® shows promise in breaking the cost barrier to the administration of PCB and in enabling compassionate, humane, high-quality care to women undergoing an MVA.

## Data Availability

The raw data supporting the conclusions of this article will be made available by the authors, without undue reservation.
